# Formulation of Solid Lipid Nanoparticles Loaded with Nociceptin/Orphanin FQ (N/OFQ) and Characterization in a Murine Model of Airway Hyperresponsiveness

**DOI:** 10.3390/ph15101210

**Published:** 2022-09-29

**Authors:** Davida Mirra, Giuseppe Spaziano, Renata Esposito, Debora Santonocito, Rosanna Filosa, Fiorentina Roviezzo, Gaetano Malgieri, Gianluca D’Abrosca, Pasquale Iovino, Luca Gallelli, Roberto Fattorusso, Carmelo Puglia, Bruno D’Agostino

**Affiliations:** 1 Department of Environmental Biological and Pharmaceutical Sciences and Technologies, University of Campania “Luigi Vanvitelli”, 81100 Caserta, Italy; 2 Department of Drug and Health Sciences, University of Catania, 95125 Catania, Italy; 3 Department of Science and Technology, University of Sannio, 82100 Benevento, Italy; 4 Department of Pharmacy, School of Medicine and Surgery, University of Naples Federico II, 80138 Naples, Italy; 5 Department of Health Sciences, University “Magna Græcia” of Catanzaro, 88100 Catanzaro, Italy

**Keywords:** SLNs, N/OFQ, AHR, airway inflammation

## Abstract

Asthma is characterized by chronic inflammation and a variable degree of airway hyperresponsiveness (AHR). Our previous papers documented a role for Nociceptin/Orphanin FQ (N/OFQ) and its receptor N/OFQ peptide (NOP) in AHR. Therefore, the aim of this study was to improve the bioavailability of N/OFQ by developing solid lipid nanoparticles (SLNs). N/OFQ-loaded SLNs were prepared by the Quasi Emulsion Solvent Diffusion (QESD) technique and then characterized. Brown Norway rats were sensitized to ovalbumin (OVA) and treated with an intratracheal administration of saline solution or N/OFQ-SLN. Then, 24 h after the last challenge, functional histological and molecular evaluations were performed. SLNs showed a mean diameter of 233 ± 0.03 nm, a polydispersity index (PDI) value around 0.28 ± 0.02 and a drug release percentage of 84.3. The in vitro release of N/OFQ from SLNs showed that the release of the peptide starts already after two hours of incubation. Animals receiving N/OFQ-SLN showed a significative decrease in allergen-induced AHR compared to the control group. These results showed the positive effects of N/OFQ-SLNs on the inflammatory process and on the mechanical properties of the airways, suggesting that the innovative nanotechnological approach may be therapeutically beneficial for asthmatic patients.

## 1. Introduction

Asthma is a complex heterogeneous disease characterized by variable degrees of airflow obstruction, chronic airway inflammation, airway remodeling and airway hyperresponsiveness (AHR). Asthma represents a significant public health problem for its increasing prevalence in a growing percentage of people worldwide [1,2]. The lack of a resolution therapy for their treatment makes necessary the identification of new targets with therapeutic potential [3,4].

Nociceptin/orphanin FQ (N/OFQ) is the endogenous peptide activator of the N/OFQ (NOP) receptor, which is widely expressed at central and peripheral levels [5]. Since the discovery of the NOP receptor and its deorphanization, numerous studies have highlighted within the N/OFQ-NOP receptor system some intriguing pharmacology [6,7,8]. The N/OFQ-NOP receptor system has been reported to play an important role in various central functions as well as in the periphery on the cardiovascular, renal, gastrointestinal and airway systems [9].

Our previous in vivo studies, in an animal model of allergic asthma, documented the activation of NOP receptors by N/OFQ as a beneficial event upon asthma immunopathology and AHR [10,11,12]. Moreover, our further research documented N/OFQ immunomodulatory and bronchodilator activity in healthy and asthmatic human airway tissues, suggesting its potential role in the pathogenesis of asthma. Notwithstanding this appreciable properties, the therapeutic use of N/OFQ is strongly compromised due to its low water solubility related to its peptide nature [13,14,15]. In fact, reduced protein stability during aerosolization and the pulmonary microenvironment represent major obstacles to its pulmonary administration [16,17,18]. This has spurred the development of selective NOP receptor agonists, including non-peptide molecules [6,19] and carrier systems for N/OFQ improving the therapeutic efficacy [20,21].

The widespread availability of increasingly sophisticated nanotechnology carriers has provided the possibility to overcome this obstacle, ensuring the achievement of high concentrations of the active ingredient at the site of interest, increasing its efficacy and safety [22,23,24,25]. Notably, solid lipid nanoparticles (SLNs) represent a promising alternative to conventional drug delivery systems. They are submicron-sized particles with diameters ranging from 50 to 1000 nanometers. SLNs are composed of a solid lipid matrix stabilized by surfactants in which the nature of the drug to be incorporated can be varied [26]. SLNs possess chemical–physical properties useful to improve the solubility, selectivity, efficacy, pharmacokinetics and toxicity of encapsulated active ingredients loaded in high percentages, favoring a high pulmonary deposition and reducing the amount of drug to be administered [27,28].

With the aim to improve the bioavailability and targeting abilities of N/OFQ, we developed SLNs loaded with a drug, as an active pharmaceutical ingredient, evaluating its efficacy in a validated animal model of allergic asthma [29,30].

## 2. Results

### 2.1. SLN Formulation and Characterization

Since N/OFQ is a hydrophilic compound, SLN-N/OFQ was formulated via the Quasi Emulsion Solvent Diffusion (QESD) method with some modifications. This method has been widely tested in our previous studies for the delivery of other hydrophilic molecules [22].

**Figure 1:**
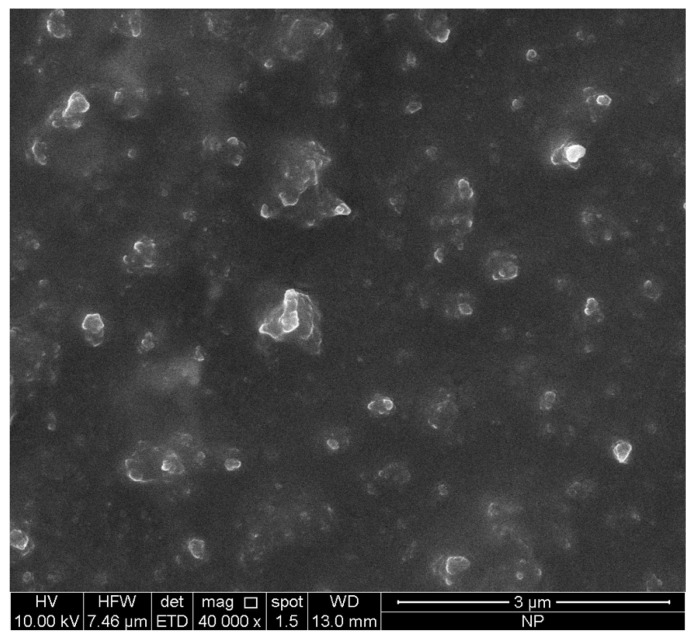
Scanning electron microscopy of N/OFQ-loaded SLNs.

As reported in Table 1, photon correlation spectroscopy (PCS) data showed good technological parameters with a homogeneity index (PDI) of 0.28 and an average particle size of 249 nm for unloaded SLN, while SLN-N/OFQ showed a PDI of 0.479 and a particle size of 240 nm; therefore, both formulations are suitable for pulmonary administration. The slight difference in terms of PDI between unloaded and N/OFQ-loaded SLN was probably due to the encapsulation of the compound, although both values are within the optimal range (PDI < 0.5). Furthermore, the nanoformulation exhibited a zeta potential (ZP) around −28 mV, predicting a good long-term stability (Figures S1 and S2). The drug loading was about 65%. Moreover, the morphology of the SLN-N/OFQ was performed using Scanning Electron Microscopy (SEM) (Figure 1). In agreement with the PCS data, the SEM images showed that the lipid nanoparticles are suitable for pulmonary administration (particle size around 250 nm) (Figure 1 and Figure S3).

### 2.2. In Vitro Release of N/OFQ from SLN

**Figure 2:**
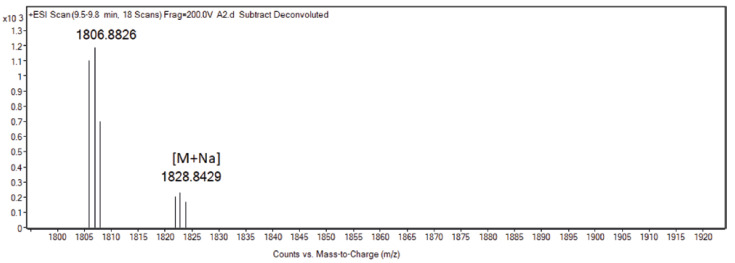
Electrospray ionization time-of-flight (ESI-TOF) deconvoluted mass spectrum of the sample after 2 h of incubation.

**Figure 3:**
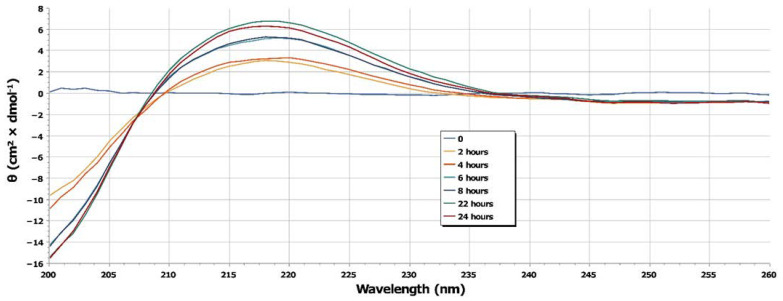
CD spectra recorded on N/OFQ samples collected from the receptor compartment at different times of incubation. Every spectrum was subtracted from the buffer contribution.

First, 2 mL of sample were collected from the receptor compartment at different incubation times and characterized by means of liquid chromatography-mass spectrometry (LC-MS) experiments to verify the presence of the released peptide. The sample collected from the receptor compartment at time 0 did not contain sufficient N/OFQ concentration to observe signals at the LC-MS instruments, while it was possible to detect the presence of the N/OFQ signal already in the sample collected after 2 h of incubation (Figure 2). Given the nature of the peptide (i.e., the absence of reliable chromophores; N/OFQ does not contain any Tyr or Trp residues) and the low concentrations at which the experiments have been conducted, we resorted to qualitatively compare the samples at different releasing times by means of circular dichroism (CD) spectroscopy (Figure 3). The CD spectra recorded as a function of time are consistent with the reported spectra of N/OFQ in water [31], thus further supporting the LC-MS results in indicating in each sample the presence of the peptide.

**Figure 4:**
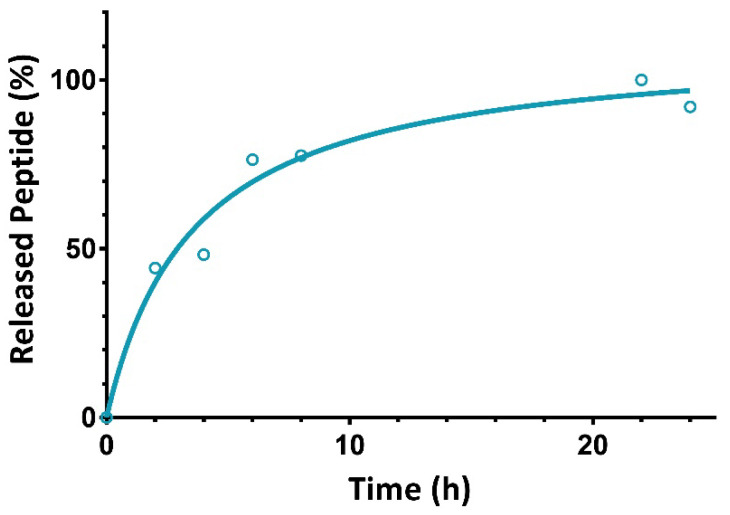
Percentage of the released peptide over time.

As it is possible to appreciate (Figure 4), the maximum peptide amount, estimated by evaluating the CD signal at 218 nm, is reached after 22 h followed by a small decrease in the concentration, thus indicating a possible plateau [32]. At 6 h of incubation, 76% of the total released amount of the peptide has been already reached.

### 2.3. Functional and Cell Count Evaluations

**Figure 5:**
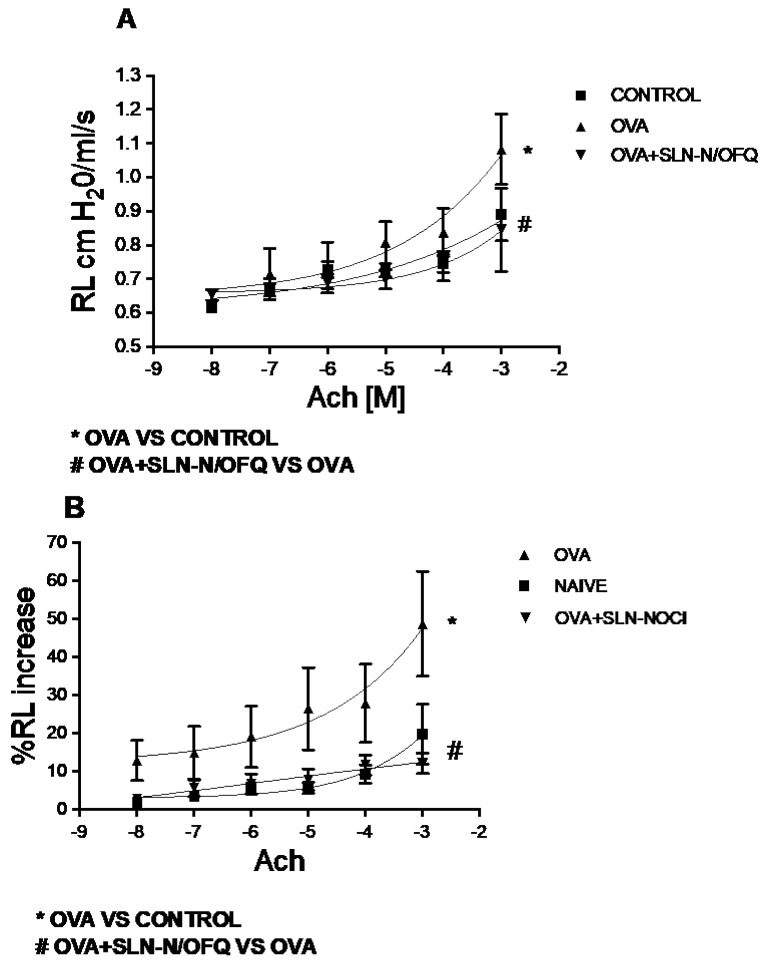
Absolute (**A**) and percentage (**B**) expression of lung resistances (RL) on ACh-induced bronchoconstriction. Endotracheal pretreatment of N/OFQ-SLNs causes a significant reduction in ACh-induced bronchoconstriction with respect to vehicle-treated rats (**A**,**B**). Data are means ± SEM (n = 10). * *p* < 0.05.

Ovalbumin (OVA)-sensitized animals that were not treated with N/OFQ-SLN showed a significant increase in acetylcholine (Ach)-induced bronchoconstriction. Endotracheal pretreatment with the N/OFQ-SLNs 30 min prior to allergen administrations caused a significant reduction in allergen-induced hyperreactivity to ACh (*p* < 0.05), restoring normal lung function (Figure 5).

**Figure 6:**
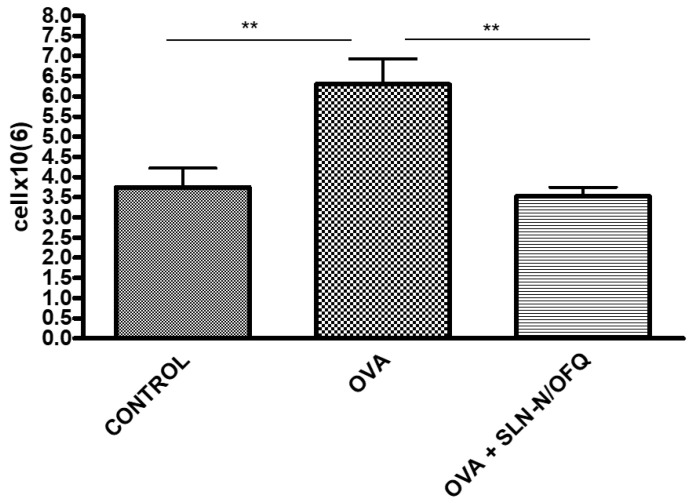
Effect of N/OFQ-SLNs on inflammatory cells. In the bronchoalveolar lavage (BAL) fluid, samples treatment with N/OFQ-SLNs significantly reduces the total number of inflammatory cells compared to the control group. Data are means ± SEM (n = 10). ** *p* < 0.01.

**Figure 7:**
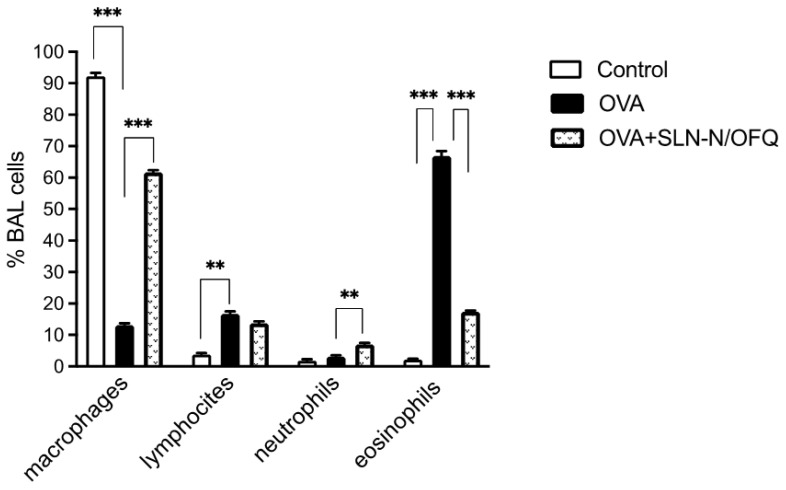
Effect of N/OFQ-SLNs on inflammatory cells. In the BAL fluid, samples treatment with N/OFQ-SLNs reduces significantly the percentage of eosinophils and increases the percentage of macrophages, compared to the control group. Data are means ± SEM (n = 10). ** *p* < 0.01, *** *p* < 0.001.

Moreover, OVA sensitization induced a significant increase in total inflammatory cell numbers (*p* < 0.01) that was significantly reduced by essential thrombocythaemia (e.t.) treatment with N/OFQ-SLNs (*p* < 0.01), returning the total cell count to values comparable to those of the control group (Figure 6). Notably, e.t. treatment with N/OFQ-SLNs showed a less prominent grade of inflammation in the proportion of lymphocytes, polymorphonucleated cells, eosinophils and macrophages, which were all cells significantly involved in asthma pathogenesis (Figure 7).

## 3. Discussion

For the first time, in the present study, we reported positive effects of N/OFQ encapsulated in SLNs on the inflammatory process and mechanical properties of the airways.

In our previous study, we demonstrated that intraperitoneally, N/OFQ treatment mitigating inflammation, airway remodeling and oxidative stress protect the murine airway against allergic asthma OVA-induced damages [10,11,12,13]. Indeed, the OVA sensitization model is the most frequently used to reproduce hallmarks of human AHR involving the infiltration of eosinophils, mast cells, neutrophils, and lymphocytes [29,30]. However, the peptide nature of N/OFQ limits its bioavailability and clinical efficacy. Therefore, looking for a carrier system for N/OFQ could solve these problems and be a good strategy for asthma treatment.

For this purpose, SLNs have been investigated as suitable inhaled drug delivery [33].

SLNs can be used to deliver treatments for a variety of diseases; in particular, SLNs have been considered for pulmonary delivery [34,35,36]. Their advantages are sustained drug release, biocompatibility and biodegradability, lower toxicity and better stability compared to previously formulated particle systems. The use of SLNs for pulmonary applications is able to produce an increase in the local drug concentration but also a reduction in the systemic side effects. Therefore, their use can achieve higher bioavailability for systemic therapies [37]. In particular, SLNs reduce the toxicity of drugs, increase the solubilities of hydrophobic drugs, and improve control over drug release [31,38,39].

SLN-N/OFQs were prepared with Lutrol F68 as surfactant and Softisan100 (Hydrogenated Coco-Glycerides) as lipid. We decided to use these materials after a screening to identify the most suitable components for the incorporation of N/OFQ. In particular, we chose Softisan 100 to prepare the lipid nanoparticles, since it is characterized by a low melting point (35 °C); this feature allows the use of a low temperature, avoiding the thermal degradation of the N/OFQ. Furthermore, in preliminary studies, it has shown that these materials had a high affinity toward N/OFQ. In our work, we encapsulated N/OFQ in SLNs, showing DPI, diameter, and zeta potential values suitable for the pulmonary delivery of N/OFQ, ensuring the good long-term stability of the system. In addition, the small size of the SLNs ensures a large surface area that allows water molecules to surround the particles, increasing the solubility of the peptide compound in biological fluids [40,41].

N/OFQ-SLNs endotracheally administered in a rat model of OVA-induced AHR were significantly effective to alleviate inflammatory process and to improve the mechanical properties of the airways, suggesting the endotracheal administration of N/OFQ-SLNs as an excellent inhaled drug delivery in the treatment of pulmonary diseases for its improved sustained release properties and targeting abilities. Indeed, the administration of the endotracheal N/OFQ-SLNs system prior to OVA sensitization restored normal pulmonary reactivity, significantly reducing pulmonary resistances, probably through the following mechanisms:

(1) N/OFQ encapsulation into SLNs enhanced the lung bioavailability of N/OFQ and wielded a profound influence on the pharmacokinetics of the drug as documented by our LC-MS analysis, which estimated a significant release of the drug (76%) already after 6 h of incubation, (2) endotracheal administration of N/OFQ-SLNs resulted in a higher accumulation of N/OFQ in lungs so that more drugs reached the inflamed tissue to exert their therapeutic effect; this hypothesis is supported by the size of the nanoparticles (250 nm) obtained through SEM images, which describe their ability to selectively deposit in peripheral airways and alveoli.

Chuanfeng et al. testified that curcumin loaded in SLNs represses the AHR and inflammatory infiltration in the treatment of asthma and improves its drug bioavailability, not only improving the drugs’ solubility in nanoformulations but also their bioavailability [42]. Wang et al. [43] also reported that curcumin–SLN administration is more effective than curcumin in attenuating asthma progression. Additionally, other authors have proven that proanthocyanidins–SLNs allow them to play a better role in oxidative stress, suppressing airway epithelial cells [44,45]. Together, these studies indicate the potential of SLNs to improve therapeutic drug efficacy as a drug delivery vehicle. 

Thus, the sustained release properties and targeting ability obtained with the innovative nanotechnology (SLN) system could be therapeutically useful for asthmatic patients due to the enhancement of pharmacological effects. Therefore, it is possible to reach a reduction and/or optimization of the dose and frequency of drug administration for achieving the minimum effective concentration, and limited distribution of the therapeutic agent to other areas, reducing the associated side effects and promoting greater patient adherence to therapy. In this way, it will be possible to optimize the therapeutic benefits of N/OFQ on inflammation and airway mechanical properties in asthma.

## 4. Materials and Methods

### 4.1. SLN Formulation

SLN-N/OFQ were prepared by the QESD method with some modifications [22]. Briefly, N/OFQ (0.08 mg) was added to 5 mL of water heated to 45 °C. The obtained solution was dispersed to lipid phase (45°) composed by Softisan 100 (0.816 g), ethanol (18 mL) and water (4 mL) using a high shear homogenizer (UltraTurrax T25; Darmstadt, Germany) at 15,000 rpm for 5 min. Thus, the obtained dispersion was slowly added to hot (45 °C) surfactant solution (Lutrol F68 7.8% *w/v*). Then, the quasi-emulsion was cooled in an ice bath for 5 min under high-speed homogenization.

### 4.2. SLN Characterization

PCS was employed to determine the Z-ave, PDI and ZP. Analyses were performed at 20 °C using a Zetasizer Nano ZS ZS90 (Malvern Instrument Ltd., Worcs, England) with a detection angle of 90° and a He–Ne laser of 4 mW at 633 nm. Before measurements, each sample was 10-fold diluted with deionized water. All measurements were taken in triplicate.

### 4.3. Scanning Electron Microscopy

SLN-N/OFQ morphology was performed using Scanning Electron Microscopy (SEM; FEI Quanta 200 SEM, Eindhoven, The Netherlands). The acceleration voltage was set at 5–20 kV. A drop of formulation was deposited onto aluminum SEM stubs. After drying, the specimen was sputter-coated with a 10 nm thick gold palladium alloy.

### 4.4. Determination of Drug Loading

The determination of N/OFQ loading in the lipid nanoparticles was performed through the tangential ultra-filtration system (Millipore) using a Pellicon XL (MWCO 1,000,000 Da). An amount of retained material was analyzed by High-Performance Liquid Chromatography (HPLC) to quantify the drug content. Chromatographic analysis was performed on a Phenomenex Kinetex C18 analytical column (50 mm × 2.1 mm, 2.1 µm). Isocratic elution was carried out using 0.1% trifluoroacetic acid (TFA) as a mobile phase, which was purged with helium for 30 min. The elution flow rate was set at 0.2 mL/min.

### 4.5. In Vitro Release Study

The release of N/OFQ was evaluated using a pre-hydrated dialysis membrane (Spectra/Por 3 Dialysis Membrane, MWCO 3.5 kD). The bag was loaded with 5 mL of SLN-N/OFQ formulation, while the receptor compartment is a beaker filled with water (40 mL) and placed in a bath at 37 °C, under magnetic stirring. Then, 2 mL of sample were taken from the receptor compartment every 2 h for 24 h (0, 2, 4, 6, 8, 22 and 24 h) and replaced with the same volume of water. Each experiment was run in duplicate.

### 4.6. In Vitro Release Profile of N/OFQ from SLN by LC-MS

Liquid chromatography–mass spectrometry (LC-MS) analyses were performed on a LC-MS Thermo Finnigan with an electrospray source (MSQ) on a Phenomenex Jupiter 5 m C18 (300 Å, 150 mm × 460 mm) column with a flow rate of 0.250 mL min^−1^ at room temperature. Samples were analyzed by LC-MS on a LC-MS Agilent Technologies 6230 ESI-TOF on a Phenomenex Jupiter 3μ C18 (150 mm × 2.0 mm) column with a flow rate of 0.2 mL min^−1^ and with a gradient of CH_3_CN (0.1% TFA) in H_2_O (0.1% TFA) from 5 to 50% in 20 min.

### 4.7. N/OFQ

Calculated mass (Da): [M+H]^+^ = 1806.2432; [M+2H]^2+^ = 904.1216;

Found (Da): [M+H]^+^ = 1806.8826; [M+1H]^1+^: 1807.8826; [M+2H]^2+^: 904.4106;

Circular dichroism spectra were recorded using a JASCO J-815 CD spectropolarimeter equipped with Peltier temperature control. Data were collected with a bandwidth of 1 nm, a data pitch of 1 nm, and a scanning speed of 50 nm/min using a quartz cuvette with a 0.1 cm path length in the 200–260 nm wavelength range. Two duplicates were acquired for every measurement, and all the spectra were subtracted from the buffer contribution.

### 4.8. Animal Study

The experimental protocol was approved by the Animal Care and Use Committee of the University of Campania “Luigi Vanvitelli” (828/2019-PR 06.12.2019). Animal care complied with the EU guidelines (2010/63/EU). The experiments were performed on n. 30 outbred male Brown Norway (Charles River Laboratories, Milan, Italy), body mass 230–250 g, housed in the University of Campania “Luigi Vanvitelli” Animal Facility, in standard cages, two animals per cage. Food and water were supplied ad libitum. Room temperature was set at 21–23 °C, 50–60% of relative humidity, and the day/night cycle was 12 h/12 h. For 7 days before initial treatment, rats were acclimated.

### 4.9. Sensitization and Treatment

We used a rat model of asthma developed by OVA (lyophilized powder, purity ≥98% by agarose gel electrophoresis, Sigma-Aldrich, St. Louis, MO, USA), sensitization and inhalation. On days 0 and 7, rats were systemically sensitized with an intraperitoneal injection of 1 mL alum-precipitated Ag in Phosphate-Buffered Saline (PBS) which contained 10 μg of OVA mixed with 3.3 mg aluminum hydroxide. Three weeks after the sensitization, the rats were placed in a perspex exposure chamber and were exposed to aerosolized OVA (1% in PBS) for 30 min/day on 3 consecutive days. The aerosol was delivered by a De Vilbiss nebulizer (De Vilbiss Health Care Ltd, Tipton, UK). The rats in the control group received the same volume of PBS. Vehicle or SLN-N/OFQ (1 μM; 15 μg/kg) (100 μL) (purity ≥ 98% by HPLC, Calbiochem Sigma-Aldrich, Darmstadt, Germany) was administered endotracheally, on days 0 and 7, 30 min before each allergen injection.

### 4.10. Experimental Design

The rats were randomly grouped as follows: control group, receiving subcutaneous injection of vehicle on days 0 and 7, sacrificed after 24 days (n = 10); OVA group, rats receiving a subcutaneous injection of the allergen OVA on days 0 and 7, challenged with OVA from day 21 to 23 and sacrificed at day 24 (n = 10); OVA + SLN-N/OFQ group, rats receiving a subcutaneous injection of OVA at day 0 and 7 and treated with an endotracheal (e.t.) administration of N/OFQ-loaded SLNs (1 μM; 15 μg/kg) (100 μL), challenged with OVA from day 21 to 23 and sacrificed at day 24 (n = 10). The rats were sacrificed to perform functional and cellular evaluations.

**Figure 8:**
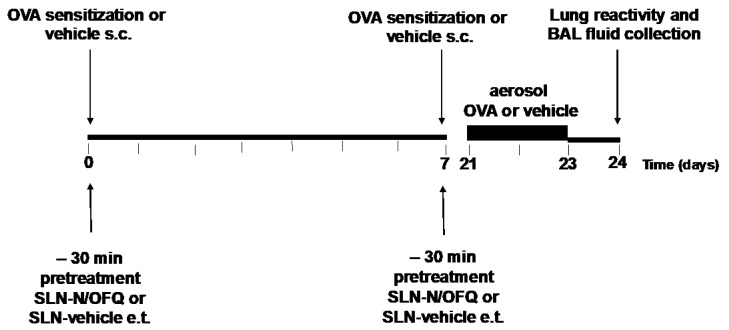
Experimental protocol.

The protocol for the sensitization, challenge and drug administration is summarized in Figure 8.

### 4.11. Measurement of Airway Hyperresponsiveness (AHR)

Lung reactivity was assessed in an isolated and perfused lung system for rat (IPL-2), Hugo Sachs Elektronik—Harvard Apparatus, as described in detail previously [46,47,48].

The animals were anesthetized with ketamine hydrochloride (100 mg/kg) (Akorn, Lake Forest, IL, USA) and medetomidine (0.25 mg/kg) (OrionPharma, Espoo, Finland) intraperitoneally administered. Registration of hyperresponsiveness (AHR) parameters in rats was performed by using a Hugo Sachs Electronik Haemodyn (Harvard Apparatus GmbH, March, Germany). All data were transmitted to a computer and analyzed with Pulmodyn software (Hugo Sachs Elektronik, March, Germany) through the following formula: P = V·C − 1 + RL·dV·dt − 1, where P is chamber pressure, C is pulmonary compliance, V is tidal volume, and lung resistance is RL. Successively, the lung resistance value registered was corrected for the resistance of the pneumotachometer. Successively, a repetitive dose–response curve to acetylcholine chloride (Ach HCl, purity ≥ 99% by TLC, Sigma-Aldrich, St. Louis, MO, USA, 10^−8^ to 10^−3^ mol/L) in all experimental groups was obtained. The dose–response curve of Ach was administered as bolus.

### 4.12. Preparation and Analysis of Bronchoalveolar Lavage Fluids (BALF)

Then, 24 h following the last allergen challenge, animals were anesthetized with a ketamine (100 mg/kg) and medetomidine (0.25 mg/kg) intraperitoneal injection (i.p.).

A 16-gauge cannula was inserted into the trachea, and lungs were gently rinsed and aspirated three times with 2 mL of sterilized normal saline containing 2% bovine serum albumin (BSA). The collected lavage fluid was centrifuged at 1000× *g* at 4 °C for 10 min. The supernatants were harvested and stored at −80 °C. The pellets were resuspended in 1 mL PBS for cell count and classification.

### 4.13. Total and Differential Cell Count

Total cell count (TCC) was performed using the Countess automated cell counter (Invitrogen). We expressed TCC as total number of recovered cells (×10^6^). The differential cell count was evaluated in light microscopy on Diff-Quik (Reagena, Italy) stained cytospin. Three hundred cells were counted for differential cell count analysis.

### 4.14. Statistical Analysis

Data are presented as mean ± SEM. All normally distributed data were analyzed by one-way analysis of variance (ANOVA) followed by Bonferroni post hoc test adjustment for multiple comparisons [49]. Post-test adjustments consider the potential error introduced as a consequence of multiple comparisons [50]. GraphPad Prism 9.0 was used for all statistical analyses. Values of *p* < 0.05 were considered significant.

## 5. Conclusions

In conclusion, due to the structural formulation and technological characteristics, which confer a high and proven safety profile, SLNs could represent an excellent strategy for the administration of N/OFQ, nominating them as potential regulators of AHR and bronchial inflammation in inflammatory airway diseases, offering an additional approach to the treatment of asthma.

## Figures and Tables

**Table 1:** Characterization of the SLN-N/OFQ formulation: the values of Z-Ave, PDI and ZP for unloaded and SLN-N/OFQ recorded at 20 °C.

Formulation	Z-Ave (nm ± SD)	PDI (-) ± SD	ZP (mV ± SD)
Unloaded SLN	248.6 ± 0.10	0.289 ± 0.16	−27.6 ± 0.32
SLN-N/OFQ	239.6 ± 0.12	0.479 ± 0.18	−29.5 ± 0.30

## Data Availability

Data is contained within the article and Supplementary Materials.

## Supplementary Materials

The following supporting information can be downloaded at: https://www.mdpi.com/article/10.3390/ph15101210/s1; Figure S1: DLS curves of unloaded SLN; Figure S2: DLS curves of SLN-N/OFQ; Figure S3: Empty SLN.
