# Supplementary material for: Formulation of Solid Lipid Nanoparticles Loaded with Nociceptin/Orphanin FQ (N/OFQ) and Characterization in a Murine Model of Airway Hyperresponsiveness
Source: Pharmaceuticals (Basel). 2022 Sep 29;15(10):1210. doi: 10.3390/ph15101210 (PMC9609590; doi:10.3390/ph15101210)
Supplement: Supplementary file 1 [file pharmaceuticals-15-01210-s001.zip › pharmaceuticals-1942553-supplementary.pdf]

Supplementary figures.

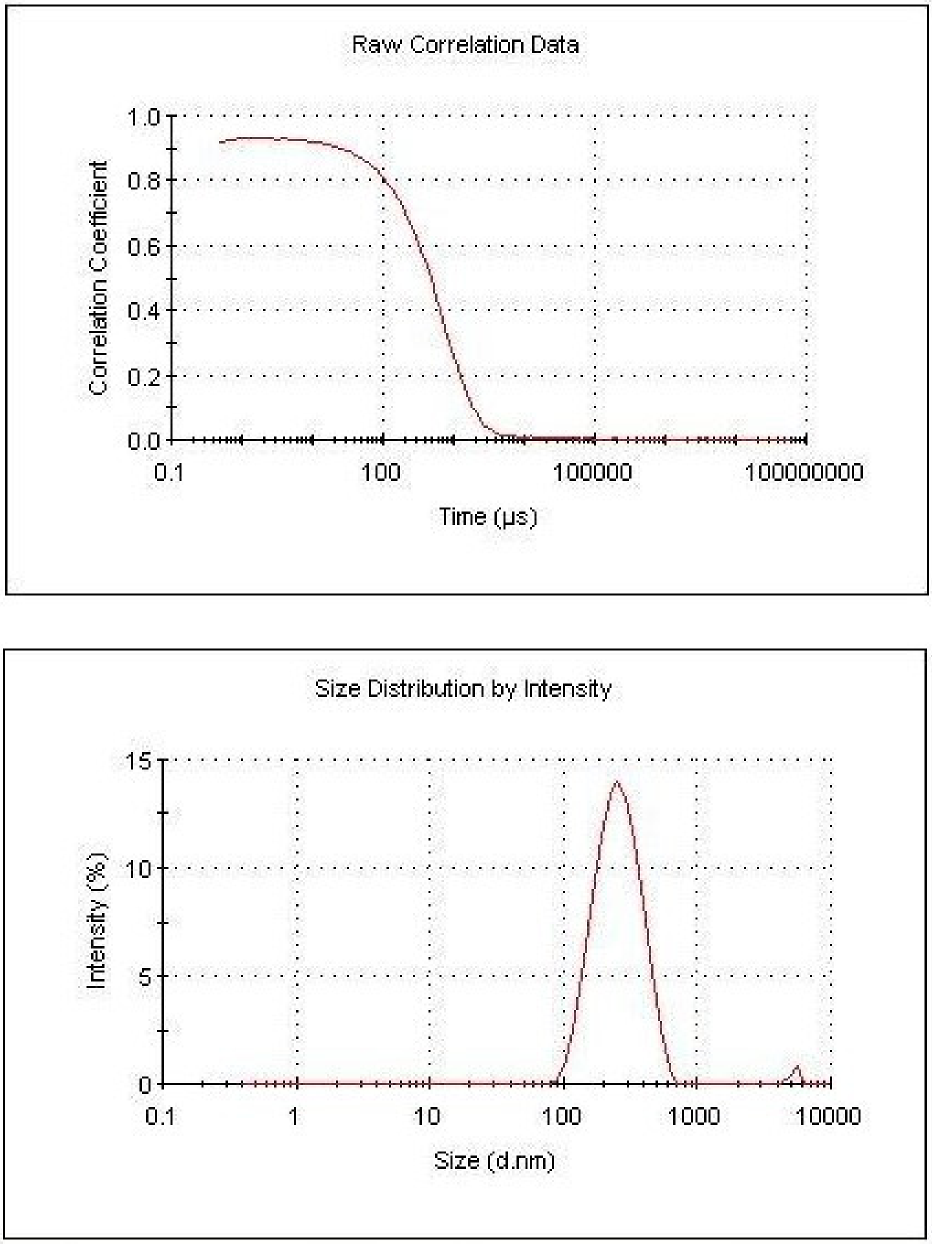

Figure S1. DLS curves of unloaded SLN.

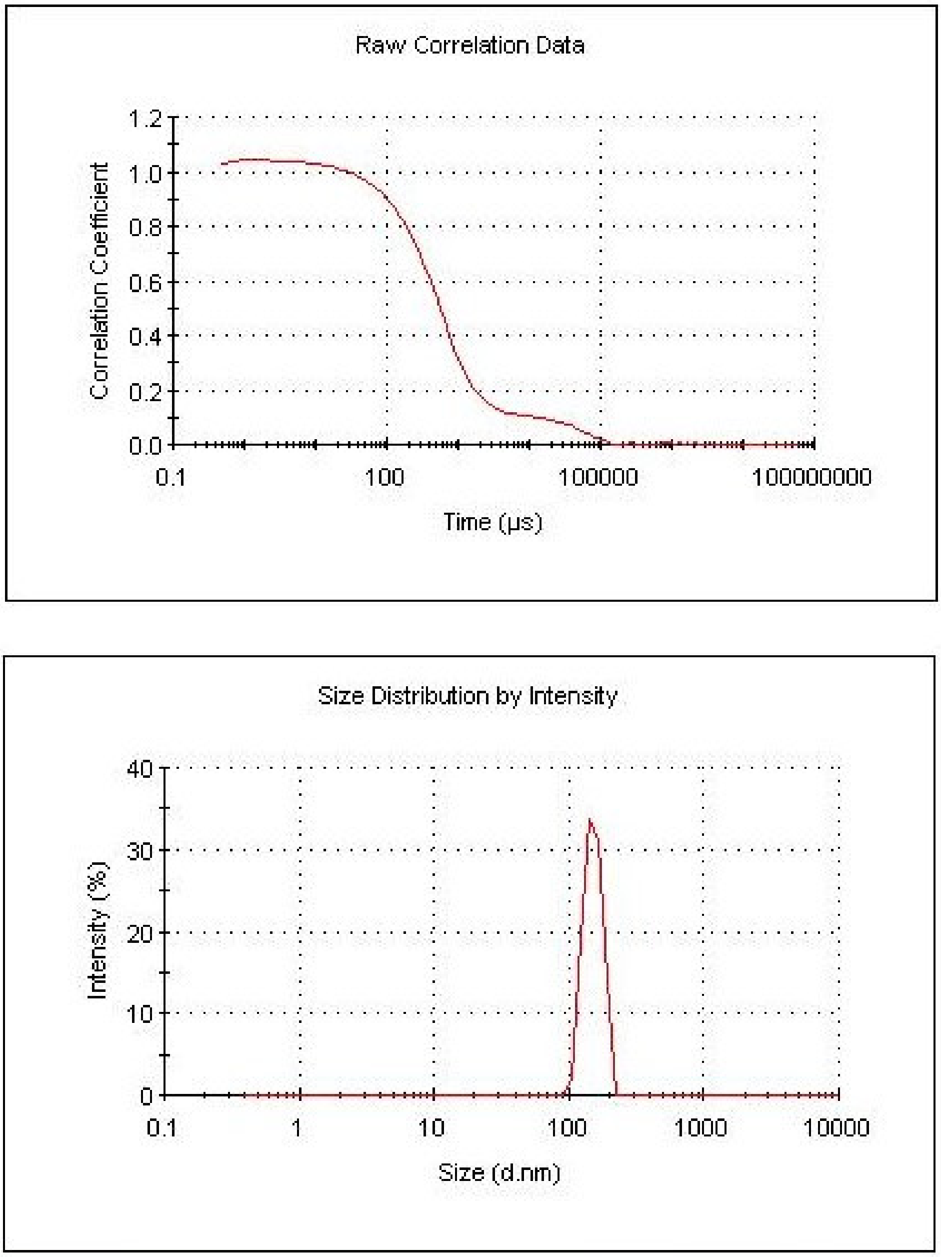

Figure S2. DLS curves of SLN-N/OFQ.

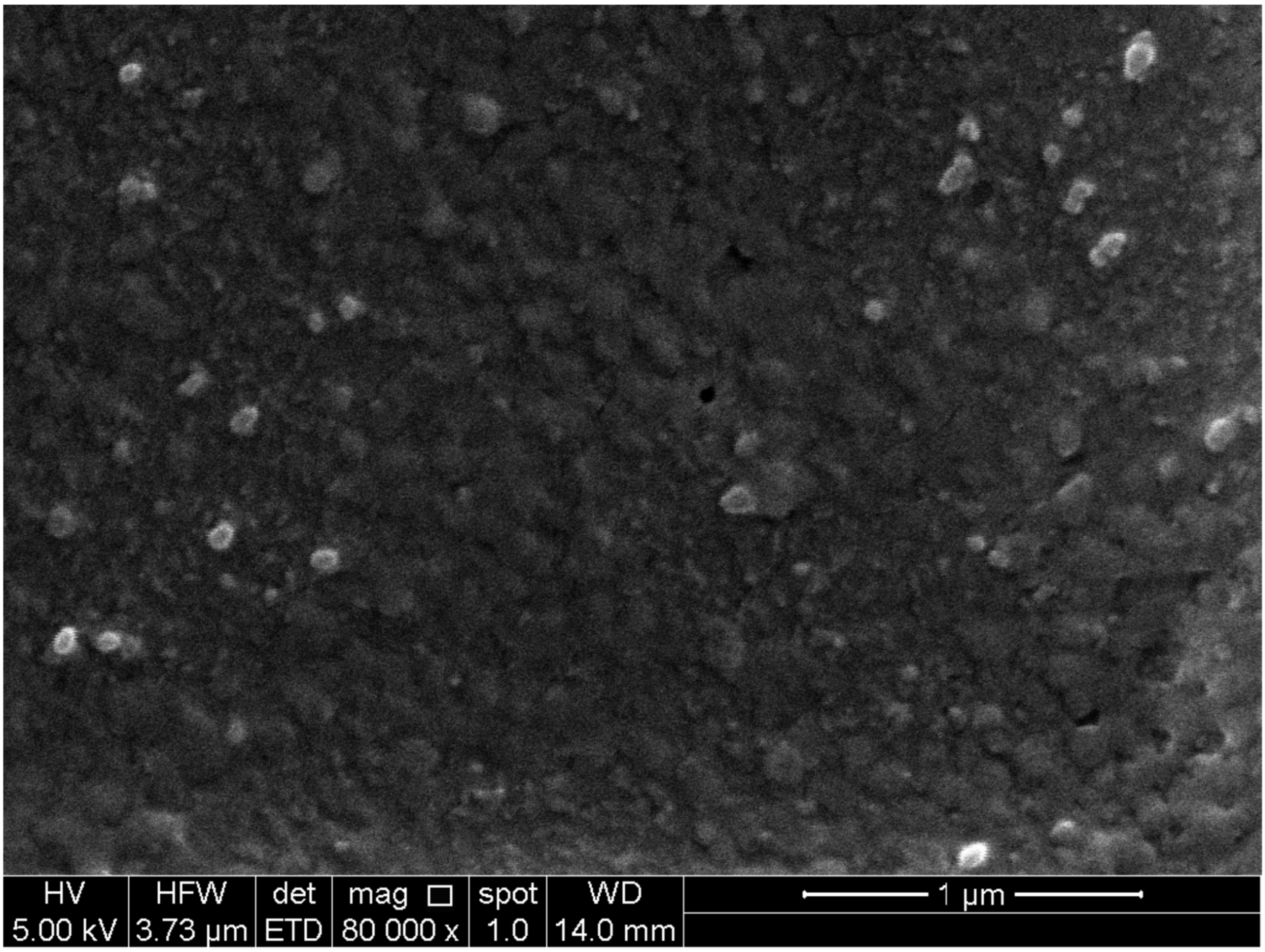

Figure S3. Empty SLN.
